# Characterization of SIPs-type aquaporins and their roles in response to environmental cues in rice (*Oryza sativa L.*)

**DOI:** 10.1186/s12870-024-05002-x

**Published:** 2024-04-22

**Authors:** Miao Miao, Ximiao Shi, Xiangzi Zheng, Binghua Wu, Ying Miao

**Affiliations:** 1 Fujian Provincial Key Laboratory of Plant Functional Biology, College of Life Sciences, Fujian Agriculture & Forestry University, Fuzhou, China; 2 College of Horticulture, Fujian Agriculture and Forestry University, Fuzhou, 350002 China

**Keywords:** Rice, AQPs, OsSIPs, Endoplasmic reticulum, Transport, Stress responses, Expression profiling

## Abstract

**Background:**

Aquaporins (AQPs) facilitate water diffusion across biological membranes and are involved in all phases of growth and development. Small and basic intrinsic proteins (SIPs) belong to the fourth subfamily of the plant AQPs. Although SIPs are widely present in higher plants, reports on SIPs are limited. Rice is one of the major food crops in the world, and water use is an important factor affecting rice growth and development; therefore, this study aimed to provide information relevant to the function and environmental response of the rice *SIP* gene family.

**Results:**

The rice (*Oryza sativa L. japonica*) genome encodes two SIP-like genes, *OsSIP1* and *OsSIP2*, whose products are predominantly located in the endoplasmic reticulum (ER) membrane but transient localization to the plasma membrane is not excluded. Heterologous expression in a yeast aquaglyceroporin-mutant *fps1Δ* showed that both OsSIP1 and OsSIP2 made the cell more sensitive to KCl, sorbitol and H_2_O_2_, indicating facilitated permeation of water and hydrogen peroxide. In addition, the yeast cells expressing OsSIP2 were unable to efflux the toxic methylamine taken up by the endogenous MEP permeases, but OsSIP1 showed subtle permeability to methylamine, suggesting that OsSIP1 may have a wider conducting pore than OsSIP2. Expression profiling in different rice tissues or organs revealed that *OsSIP1* was expressed in all tissues tested, whereas *OsSIP2* was preferentially expressed in anthers and weakly expressed in other tissues. Consistent with this, histochemical staining of tissues expressing the promoter-β-glucuronidase fusion genes revealed their tissue-specific expression profile. In rice seedlings, both *OsSIPs* were upregulated to varied levels under different stress conditions, including osmotic shock, high salinity, unfavorable temperature, redox challenge and pathogen attack, as well as by hormonal treatments such as GA, ABA, MeJA, SA. However, a reduced expression of both *OsSIPs* was observed under dehydration treatment.

**Conclusions:**

Our results suggest that SIP-like aquaporins are not restricted to the ER membrane and are likely to be involved in unique membrane functions in substrate transport, growth and development, and environmental response.

**Supplementary Information:**

The online version contains supplementary material available at 10.1186/s12870-024-05002-x.

## Background

Water is fundamental for life and is an indispensable element of living organisms. Living beings have developed numerous protein channels that are integrated into their membranes to facilitate the movement of water across the body during different physiological processes [1, 2]. Aquaporins (AQPs) are proteins situated in biological membranes that transport water. AQPs are members of the major intrinsic proteins (MIPs) and are prevalent among microorganisms, plants, and animals [3].

AQPs are low molecular weight proteins, approximately 23–31 kDa, and their sequence-to-structure conservation is significant [4]. In addition to conducting water, AQPs are channels for small uncharged solutes such as CO_2_, H_2_O_2_, glycerol, NH_3_ and urea, as well as metalloids such as silica, selenite, arsenite and boric acid [5–10]. Typical AQPs monomers consist of six transmembrane (TM) α-helices and two half-transmembrane α-helices, which are folded into monomers in an inverted front-backward manner. These monomers exist as tetramers on the membrane, but each one functions as an independent channel [5, 11–13]. The conducting pore of each AQP is formed mainly by hydrophobic amino acid residues, and there are two critical constrictions, the asparagine-proline-alanine NPA regions in the center and the aromatic-arginine (ar/R) constriction at the extracellular entrance [5, 11–13]. The NPA region is critical for water or solute transport, together with the ion repulsion mechanism, and is formed by the convergence of two NPA residues conserved in the sequence located at the center of the membrane. The ar/R constriction, which comprises four amino acid residues, determines whether the channel is a strict water channel or a broadly selective solute channel bond and serves as the substrate screening region of the channel [5, 11–13]. As substrates enter the internal AQPs, favorable interactions between water and amino acid residues on the surface of the pore decrease, and solute-amino acid residue interactions take over; the stronger the interaction, the easier it is for the solute to pass through [10, 14] .

To adapt to a variety of external environments, plants have evolved a vast number of AQP members over the course of their extensive evolutionary process, surpassing that of animals [15]. Plants possess a large family of AQPs that can be divided into seven distinct subfamilies: Plasma membrane intrinsic proteins (PIPs); Tonoplast intrinsic proteins (TIPs), Nod26-like intrinsic proteins (NIPs), Small and basic intrinsic proteins (SIPs), GlpF-like intrinsic proteins (GIPs), Uncategorized X intrinsic proteins (XIPs), and Hybrid intrinsic proteins (HIPs) have been studied previously [16–19]. It has been demonstrated that plant AQPs are widely expressed throughout plant tissues, including roots, stems, leaves, flowers, fruits and seeds [4, 15, 20–22]. These proteins are essential for various physiological processes, including water regulation, ionic balance maintenance, photosynthetic metabolism, nutrient absorption, growth and development, antistress response, and signal transduction in plants [4, 15, 20–22]. Despite many studies on the transport activity, function, and regulation of the members of the PIPs, TIPs, and NIPs, the SIP subfamily has received comparatively little attention.

SIPs in plants show a high degree of sequence similarity to the mammalian AQP11, and both are localized to the endoplasmic reticulum [23–25]. Three isoforms of AtSIPs have been identified in *Arabidopsis*, namely AtSIP1;1, AtSIP1;2, and AtSIP2;1. Among them, only AtSIP1;1 and AtSIP1;2, have demonstrated water permeability through stopped-flow measurements by heterologous expression in yeast [24]. The first NPA motifs in AtSIP1;1, AtSIP1;2, and AtSIP2;1 are replaced with NPT, NPC, and NPL, respectively. This alteration can potentially impact their solute permeability [23–25]. Furthermore, there is evidence indicating that AtSIP1;1 supports hydration of pollen on the pistil whereas AtSIP2;1 contributes to germination and elongation of pollen tube in Arabidopsis [26, 27]. VvSIP1;1 in grape has also been demonstrated to facilitate water transport through the endoplasmic reticulum membrane [28]. The overexpression of the soybean *GmSIP1;3* gene in tobacco exhibited growth retardation and tolerance to hydrogen peroxide [29]. Overexpression of *MaSIP2;1* in banana plants enhanced their drought and cold stress resistance [30]. Rice is a crucial global food crop that has a significant impact on food security [31]. The rice genome contains two *OsSIP* genes, *OsSIP1* and *OsSIP2* [17, 32], which have not been characterized yet.

In this study, we compared the predicted structures of the rice OsSIP1 and OsSIP2 proteins to other SIPs by using bioinformatic tools, and carried out yeast phenotypic assays to determine whether OsSIPs could mediate water or other solute transport. The subcellular localization of both OsSIP1 and OsSIP2 was visualized via fusion with the Green Fluorescent Protein (GFP). The tissue expression pattern of *OsSIPs* at different developmental stages was examined through a combination of qRT-PCR and histological staining of promoter-GUS transgenic plants. Furthermore, the expression profiles of *OsSIPs* were examined under phytohormonal, biotic and abiotic stresses.

## Methods

### Plant materials and treatments

Rice Nipponbare (*Oryza sativa subsp. Japonica*) seeds were sterilized in 0.3% NaClO for 15 min, transferred to Kimura B nutrient salts solution after germination, and cultured in a greenhouse with 12-h-dark (25 °C) / 12-h-light (28 °C) cycle. After 2 days of germination, roots, stems, leaves, sheaths at different developmental stages as well as reproductive glumes and anthers were placed in liquid nitrogen immediately after harvest and stored at -80 °C for total RNA isolation.

For abiotic stresses and hormones treatments, seedlings from 2-week-old-cultured in Kimura B nutrient solution were treated with dehydration (on filter paper without water supply), 20% PEG 6000, 150 mM NaCl, 1% H_2_O_2_, 2 mM dithiothreitol (DTT), 100 µM salicylic acid (SA), 100 µM methyl jasmonate (MeJA), 100 µM gibberellin (GA3) or 100 µM abscisic acid (ABA). For temperature treatments, seedlings were transferred to a growth chamber at 4–42 °C under the same growth conditions as described above. Whole seedlings were harvested at different time points (3, 6 and 9 h) after treatment and samples were immediately frozen in liquid nitrogen for RNA isolation. The untreated 0 time point plants served as control.

In the pathogen infection treatment experiment, 3 to 4-week-old seedlings of rice were spray-inoculated with the spore suspension of *M. oryzae* strain Guy11 (5 × 10^4^ conidia/mL in 0.02% (w/v) Tween20 solution). Rice seedlings were sprayed with 0.02% (w/v) Tween 20 solution as a mock control. The inoculated plants were incubated in the dark at 20–23 °C and 80% humidity for 24 h and then in a growth chamber at 25 °C and 80% humidity for 5 days. Leaves were excised and pooled at different time points after inoculation for RNA isolation.

Each experiment was set up with three biological replicates (each replicate contained at least 5 different plants).

### Bioinformatics analysis

The amino acid sequences of all AQPs (*O. sativa*, *A. thaliana*, *N. tabacum*, and *H. sapiens*,) were aligned using the MEGA X and a phylogenetic tree was constructed using Maximum Likelihood method based on the JTT matrix-based model with 1000 bootstraps. Then, we used EvolView online to display the phylogenetic tree (https://www.evolgenius.info). The ar/R constriction site amino acids were deduced based on sequence alignment with known aquaporin structures [6].

Homology modeling was conducted using SWISS-MODEL server [33]. Briefly, template search with BLAST and HHblits were performed against the SWISS-MODEL template library and the best template was selected to build the models based on the target-template alignment using ProMod3. The resulting SWISS-MODEL predicted structural models of OsSIP1 and OsSIP2 were visualized using the PyMol software (version 2.5). To make a superimposition of the predicted structures of OsSIP1 and OsSIP2, both PDB files were uploaded to the FATCAT server (https://fatcat.godziklab.org/fatcat/fatcat_pair.html) for pairwise structural alignment, and the aligned PDB file was downloaded and visualized using the PyMol.

The transmembrane helices of OsSIP1 and OsSIP2 were deduced from the homology models and the topology of the proteins was drawn accordingly using the PROTTER (http://wlab.ethz.ch/protter). For the prediction of potential phosphorylation sites of OsSIP1 and OsSIP2 amino acid residues, the respective protein sequence was submitted to NetPhos v 3.1 [34].

### Plasmids construction

The information of *OsSIP1* (LOC_Os01g08660) and *OsSIP2* (LOC_Os03g20410) CDS sequence and protein sequence was downloaded from the Rice Genome Annotation Project(http://rice.plantbiology.msu.edu/). For subcellular localization, the coding sequences of *OsSIP1* and *OsSIP2* were cloned into the entry vector pENTYR®-/D-TOPO respectively (Invitrogen), according to the manufacturer’s instructions, and subsequently cloned into the Gateway binary vector pK7FGW2.0 (C-terminal eGFP) or pFGFP2GW (N-terminal eGFP) driven by the cauliflower mosaic virus 35 S promoter with an LR reaction (Gateway recombination, Invitrogen). For Promoter- the β-glucuronidase (GUS) constructs, a fragment of 1642-bp or 1115-bp upstream of the first ATG of *OsSIP1* or *OsSIP2* were similarly cloned into the Gateway binary vector pHGWB433, yielding respective binary vector for *in planta* expression of the GUS reporter driven by the native promoters. For yeast growth assays, The coding sequences of OsSIP1/2-GFP fusion proteins and GFP were each subcloned into the yeast expression vector pRS426met25 with the strong promoter MET25 [35]. The primers used are listed in Table S2. All the constructs were verified by sequencing.

### RNA isolation and qRT-PCR

Total RNA was isolated from various tissues of rice and seedlings after abiotic stress treatment using Trizol reagent (Invitrogen). Extraction was performed according to the kit instructions. First-strand cDNA was synthesized using Prime Script RT Reagent Kit (TransGen Biotech, Beijing) according to the instructions. qRT-PCR was performed using TransStart Top Green qPCR SuperMix Kit (Transgen Biotech, Beijing, China) on the CFX96 Real Time System (Bio-Rad, USA) according to the manufacturer’s instructions, using specific primers as listed in Table S2. Relative expression was calculated using 2^−△△Ct^. Statistical analyses were performed using GraphPad Prism software (version 8) for Student t-test on the means of the three biological replicates and three technique replicates.

### Expression in yeast and growth assays

The *S. cerevisiae* strain *BY4742Δfps1 (MAT, his3-1, leu2Δ0, lys2Δ0, ura3Δ0, yll043w::KanMX)* lacking the endogenous aquaglyceroporin Fps1 and its isogenic parent strain BY4742 (*MAT, his3-1,leu2Δ0, lys2Δ0, ura3Δ0*) were used [36]. Plasmids pRS426met25 containing the coding sequences of *OsSIPs-GFP* or *GFP* alone were introduced into the yeast using the standard PEG protocol. We incubated the SD (-Ura, pH 5.5) plates (selection for Ura3 containing plasmid) at 28 °C for 3–5 days until transformant colonies appeared.

Different yeast transformants were grown in liquid SD-Ura medium to an OD_600_ of 1. The cultures were diluted to an OD_600_ of 1, and 10 µl of a series of four dilutions (1:10, 1:100, 1:1000, 1:10000) were spotted on different agar plates. Growth of the yeast cells was monitored after 5–7 days of incubation at 28 °C.

For yeast hyperosmotic and peroxide stress assays, the respective transformants were grown on SD (-Ura) medium containing different concentrations of (0 M, 1.5 M, 2 M) KCl, (0 M, 1.5 M, 2 M) sorbitol, or H_2_O_2_ (0 mM, 2 mM, 2.5 mM). For methylamine toxicity experiments, MES buffered (20 mM, pH 5.5) YNB-glucose medium containing 0.1% proline as the sole nitrogen source was supplemented without or with 50 or 100 mM methylammonium (MeA, Sigma).

### Promoter-GUS constructs and histochemical analysis

To generate Promoter-GUS transgenic plants, Nipponbare (*Oryza. sativa subsp. Japonica*) was transformed using *Agrobacterium*-mediated transformation, and transgenic calli were screened with 50 mg L^− 1^ hygromycin. T2 homologous transgenic lines plants were used for the GUS analysis. The primers used for cloning the promoters and constructing the promoter-GUS expression vectors, as well as for transgenic detection are listed in Table S2.

Histochemical GUS assays were conducted using methods previously described [37]. Different tissues were collected and immersed in a vacuum using GUS staining solution for one hour in the dark. The solution contained 100 mM sodium phosphate (pH 7.0), 10 mM Na_2_EDTA, 1 mM K_3_[Fe(CN)_6_], 1 mM K_4_[Fe(CN)_6_], 0.5% Triton X-100, 20% methanol, and 1.5 mg mL^− 1^ X-gluc. The samples were stained in the dark at 37 °C overnight. After incubation in an 80% ethanol solution to remove chlorophyll, the stained samples were observed with a stereomicroscope (Leica M205FA).

### Protein extraction and western blotting

Crude membrane proteins were extracted from yeast incubated at 30 °C for 16 h according to a previously described method [38]. SDS-PAGE and Western blot analysis were carried out using standard protocols [39]. Briefly, crude membrane extracts were prepared, followed by SDS-PAGE, and then the proteins were transferred to Immobilon-P PVDF membranes using Trans-Blot Transfer System (Bio-Rad). Membranes were blocked for 3 h in 5% fat-free milk in PBST and then incubated with the corresponding antibodies. Antibodies used for western blot include mouse anti-GFP (Transgene, 1:4000 dilution) and anti-mouse (Transgene, 1:5000 dilution). Bands were visualized by using ECL-Western Blot Kit (Cwbio) according to the manufacturer’s instructions. Chemiluminescent signals were acquired by using a ChemiDoc™ Touch Imaging System (Bio-Rad).

### Subcellular localization and confocal microscopy observation

The *Agrobacterium* strain GV3101 was transformed with vector expressing OsSIP tagged with either a N-terminal (pFGFP2GW-OsSIPs) or a C-terminal GFP (pK7FGW2.0-OsSIPs), or vector expressing either an ER-retained red fluorescent protein (pCAMBIA3301-RFP-HEDL) or a cytosolic fluorescent protein (pCAMBIA3301-GFP/RFP). The transformant was cultured in a solution containing 10 mM MES, 10 mM MgCl_2_, and 100 mM acetosyringone to an optical density (OD_600_) of 0.6 to 0.8. Then, two strains were mixed and incubated at the room temperature for at least 3 h. The mixture was infiltrated into 4-week-old tobacco (*Nicotiana benthamiana*) leaves, which were incubated in the dark for 24 h, followed by light culture for 12–48 h. The fluorescence signals of the leaves were detected by laser confocal microscopy. Rice protoplast isolation and transfection were performed as described [39], and in some cases, a combination of the above-mentioned vectors were used for co-transfection as indicated in Fig. 4B of the Results section.

Fluorescence imaging was realized in a Leica SP8 Confocal Microscope, with a setting for GFP (Ex = 488 nm, Em = 505 to 535 nm), for chlorophyll auto-fluorescence (Ex = 633 nm, Em = 650 to 710 nm) and for RFP (Ex = 532 nm, Em = 550 to 600 nm).

## Results

### Structural module of the two rice SIP-like proteins

**Fig. 1:**
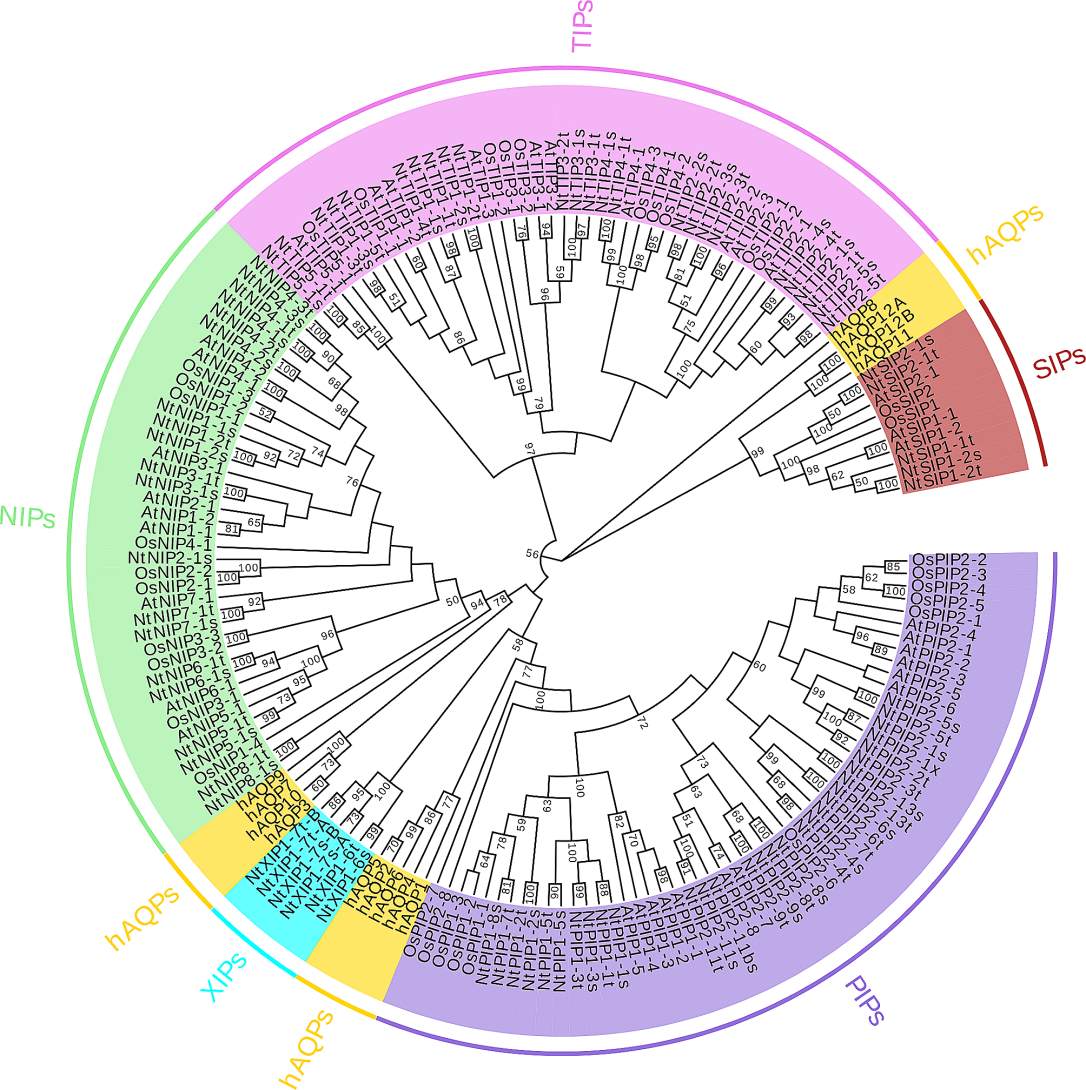
Phylogenetic analysis of 167 aquaporins, which from *O. sativa* (Os), *A. thaliana* (At), *N. tabacum* (Nt), and *H. sapiens* (hAQPs, yellow) respectively. The AQPs of plants clustered into five different subfamilies (PIPs, TIPs, NIPs, SIPs and XIPs). The full-length amino acid sequences were aligned using ClustalW and phylogenetic tree was constructed using MEGA X based on the NJ method. The numbers at the branch points represent the bootstrap values of the phylogenetic tree (1,000 replications,≥50%)

**Fig. 2:**
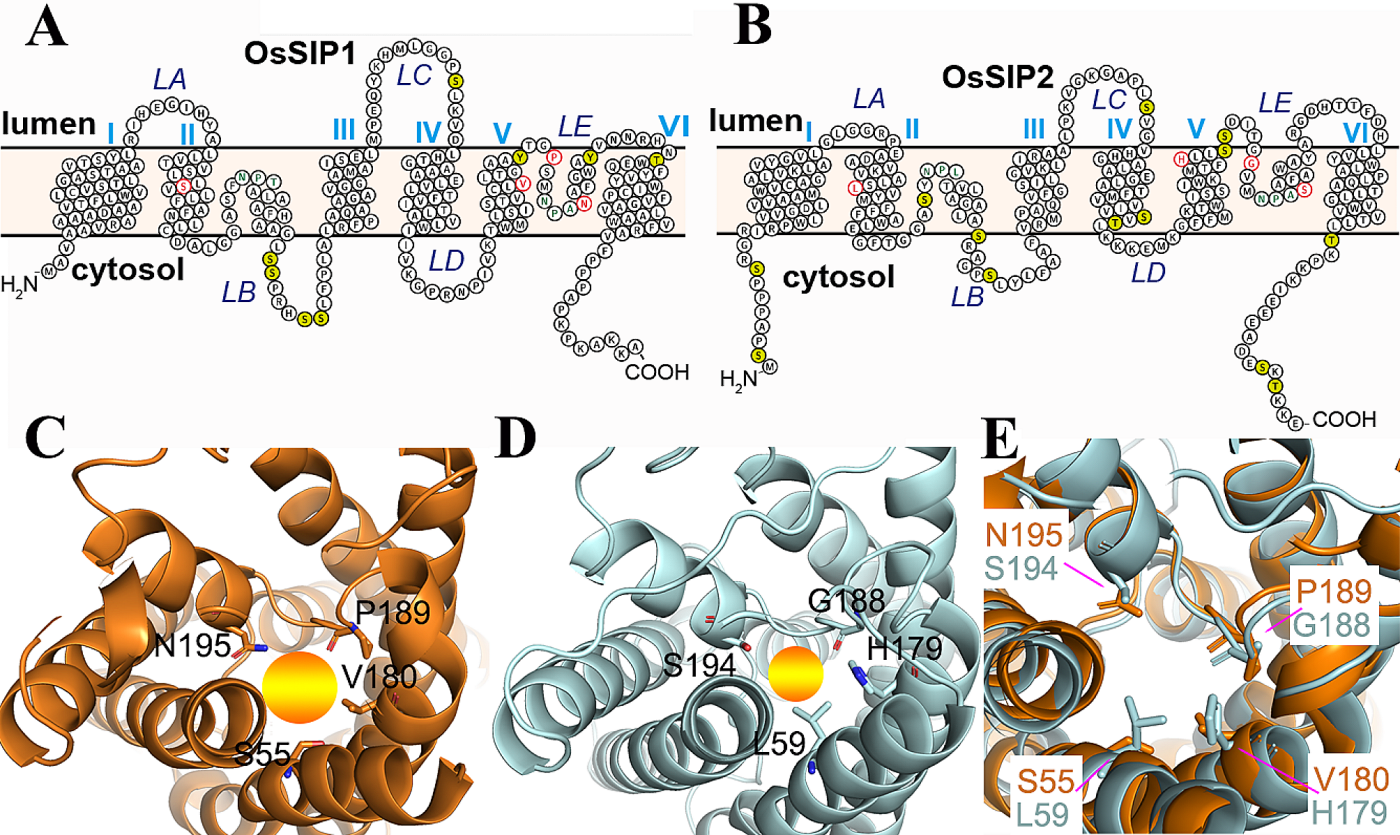
Structural modelling of OsSIP1 and OsSIP2 proteins. (**A, B**) Membrane topology of OsSIP1 and OsSIP2 The transmembrane helices are numbered in Roman numerals, and the loop regions are indicated (LA-LE). Residues in the NPA motif and the ar/R constriction (R1, R2, R3, R4) are highlighted in green and red respectfully. Residues shaded in olive green represent the putative phosphorylation residues. (**C, D**) The ar/R region in the 3D structures of OsSIP1 and OsSIP2 predicted by homology modelling using the SWISS-MODEL server and depicted using PyMol. The four residues of the region are shown as sticks for their side chains. The colored spheres represent the pore at the constriction. (**E**) A superimposition of the predicted structures of OsSIP1 and OsSIP2 showing only the ar/R constrictions. See Methods for details

Previous studies have demonstrated that the *SIP-like* in rice(*Oryza sativa*) consist of two members *OsSIP1* and *OsSIP2* [17]. By constructing evolutionary trees for multiple species and analyzing amino acid sequence homology and identity, it was revealed that they belong to two different subfamilies, SIP1s and SIP2s, respectively (Fig. 1). Homology modeling and topological analysis of OsSIP1 and OsSIP2 revealed that both have a short N-terminus, 6 transmembrane structural domains and five loops that connect the TMs. The C-terminus, N-terminus, LB, and LD loops are intracellularly held, while the LA, LC, and LE loops are anchored extracellularly, Additionally, two NPA motifs, and an ar/R-restricted region are present (Fig. 2A, B).

Unlike typical AQPs, the NPA regions conserved between TM2 and TM3 of OsSIP1 and OsSIP2 have changed to NPT and NPL, respectively. The ar/R-restricted regions, which govern their permeability to different substrates, are Serine55, Valine180, Proline189 and Asparagine195 in OsSIP1 or Leucine59, Histidine179, Glycine188 and Serine194 in OsSIP2 (Fig. 2C, D). Previous studies have confirmed the water permeability of *Arabidopsis* AtSIPs and grape VvSIP1;1 [24, 28]. Further research has found that the *Homo sapiens* hAQP11, which exhibits a closer homology to the plant SIPs family (Fig. 1), can transport water, H_2_O_2_ and glycerol [40, 41]. A comparison of the two conserved constrictions of OsSIPs with those of other SIP orthologues and other typical AQPs members that have been studied so far suggested that the OsSIP2 is likely more restricted in the pore layouts owing to the projected side chains of the Leu59 and His179 in the ar/R region (Fig. 2E). Based on the predicted structural models (Fig. 2C, D), we propose that OsSIPs may behave more like a water channel rather than a glycerol channel. Finally, putative phosphorylation sites are presented in the cytoplasmic LB loop and N/C-terminus of OsSIPs (Fig. 2A, B). As phosphorylation at several sites in the LB loop and C-terminus of AQPs has been found to control their water transport activity in plants [42–45], these predicted sites are potentially regulatory in nature.

### Phenotypical analysis of yeast cells expressing OsSIP1 and OsSIP2

**Fig. 3:**
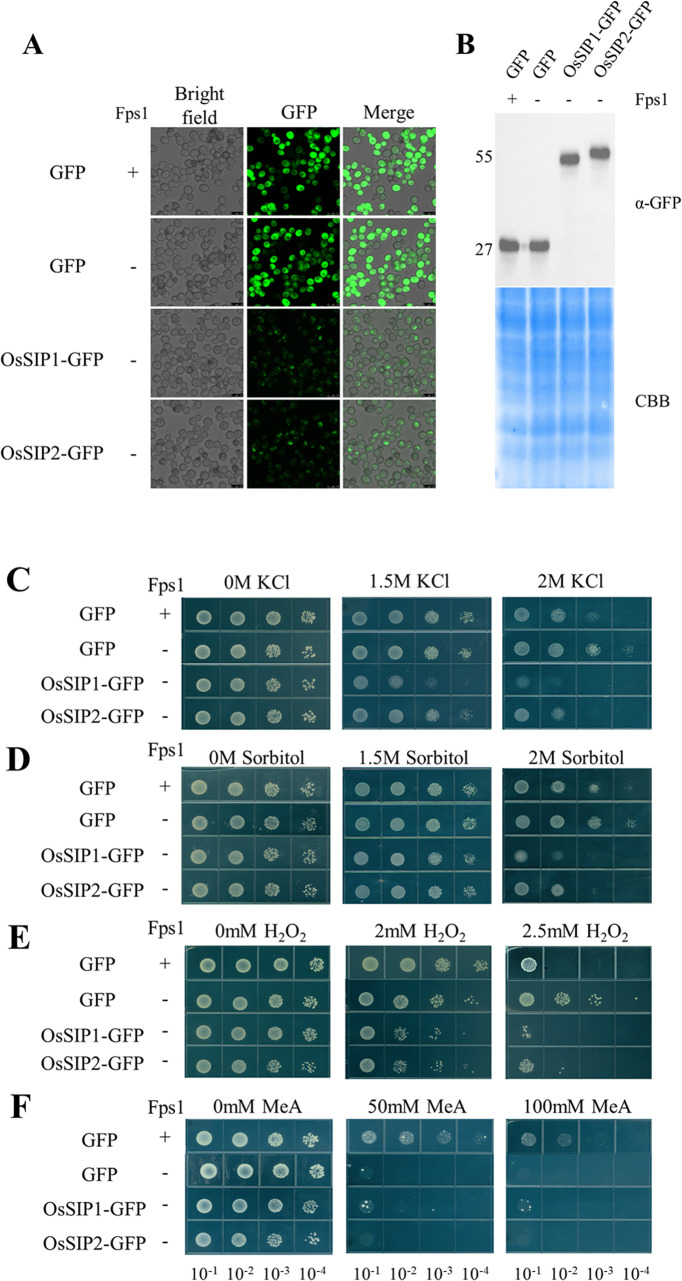
Phenotypic analysis of *OsSIP*-expressing cells of the *S. cerevisiae* BY4742 Δfps1 mutant. The wild-type BY4742 or BY4742Dfps1 mutant transformed with the pRS426met25::GFP vector was used as the positive and negative control, respectively. Expression of the recombinant OsSIP1-GFP or OsSIP2-GFP (GFP fusion at the C-terminus) fusion protein was confirmed by fluorescence imaging (**A**) and Western blot using an antibody against the GFP-tag (**B**). Growth assays were performed on SD-Ura (pH: 5.5) plates with different concentrations of the KCl, sorbitol and H_2_O_2_ (**C-E**). Ammonia permeation experiments were performed on plates in Mes- or Mops-buffered (20 mM, pH 5.5) YNB-glucose medium containing different concentrations of methylamine (MeA) with 0.1% proline added as a nitrogen source (**F**). The different yeast lines were grown in the appropriate liquid medium until the OD_600_ was 1.0, then serially diluted 10-fold and spotted onto different plates. Plates were cultured at 30 °C for 3–5 days and then imaged

A *S. cerevisiae fps1* mutant, *BY4742* (*Δfps1*), which lacks the endogenous aquaglyceroporin Fps1, was successfully utilized for functional complementation analysis to investigate the permeability of different aquaporins to several substrates [36, 46–50]. To investigate the impact of OsSIP1 and OsSIP2 on substrate transport, we evaluated the growth of yeast *BY4742 (Δfps1)* cells transformed with *pRS426met25-OsSIPs-GFP* (GFP fusion at the C-terminus) or *pRS426met25-GFP* (negative control) on plates containing the specified substrate or not. Additionally, we used *pRS426met25-GFP* transformed wild-type yeast strain BY4742 as a positive control. Fluorescence of the OsSIPs-GFP fusion proteins or GFP indicated successful expression of the proteins (Fig. 3A), which was further verified by western blot assays (representative as in Fig. 3B). The expression of OsSIP1-GFP and OsSIP2-GFP exhibited no toxicity to the yeast cells, as evidenced by comparable growth on plates without substrate addition that was identical to that of the cells transformed with the control vectors (Fig. 3).

When challenged with hyperosmotic stress imposed by high concentrations of either KCl or sorbitol, the yeast cell accumulates intracellular glycerol and closes the aquaglycerol channel Fps1 for survival, and the fps1 mutant is more resistant due to a lack of expressed water and/or glycerol channels [49]. Expression of both OsSIPs made the yeast cells more sensitive than the wild-type yeast and the fps1 mutant (Fig. 3C, D), indicating that a facilitated efflux of water could contribute to the defective growth. Thus, based on their phenotype, OsSIP1 displays higher water or glycerol permeability than OsSIP2.

To test whether the OsSIP1 and OsSIP2 could transport H_2_O_2_, growth assays were carried out on agar medium (SD-Ura, pH 5.5) with increasing concentrations of H_2_O_2_. The wild-type cell grew slightly better than the *fps1* mutant under 2 mM H_2_O_2_ but could not withstand at 2.5 mM H_2_O_2_ (Fig. 3E), consistent with the previously reported notion that oxidative stress rapidly induces Fps1 degradation at low H_2_O_2_ concentration [50]. Expression of OsSIP1 and OsSIP2 in the mutant resulted in a significant reduction of growth under increasing H_2_O_2_ concentration (Fig. 3E). Similar to the situation under hyperosmotic stress, heterologous expression of OsSIP1 seemed to cause the yeast cell to be slightly more sensitive to H_2_O_2_ than OsSIP2 expression (Fig. 3E). The sensitivity to H_2_O_2_ is presumably contributed by the SIP proteins sitting at the plasma membrane although their GFP fluorescent signal is predominant at the ER (Fig. 3E).

To determine ammonia permeation, the agar medium (SD-Ura, pH 5.5) was supplemented with the toxic ammonia analogue MeA, which was taken up by the MEP proteins and accumulated in the cytosol causing detrimental effects to the cells. Detoxification could be achieved by the presence of a functional channel that permits the release of MeA outside the cells [36]. As expected, the *fps1* mutant was unable to grow in MeA-containing medium, while cells with FPS1 allowed sustainable growth in medium with up to 100 mM MeA (Fig. 3F). The expression of OsSIP1 in the mutant yeast rescues only weak growth whereas that of OsSIP2 had no effect at all (Fig. 3F). This observation demonstrates that OsSIP1 may have limited methylamine permeability and OsSIP2 is likely a strict water channel. Another possibility was not excluded that both SIP proteins are not sufficiently localized to the plasma membrane.

### OsSIP1 and OsSIP2 subcellular localization analysis

**Fig. 4:**
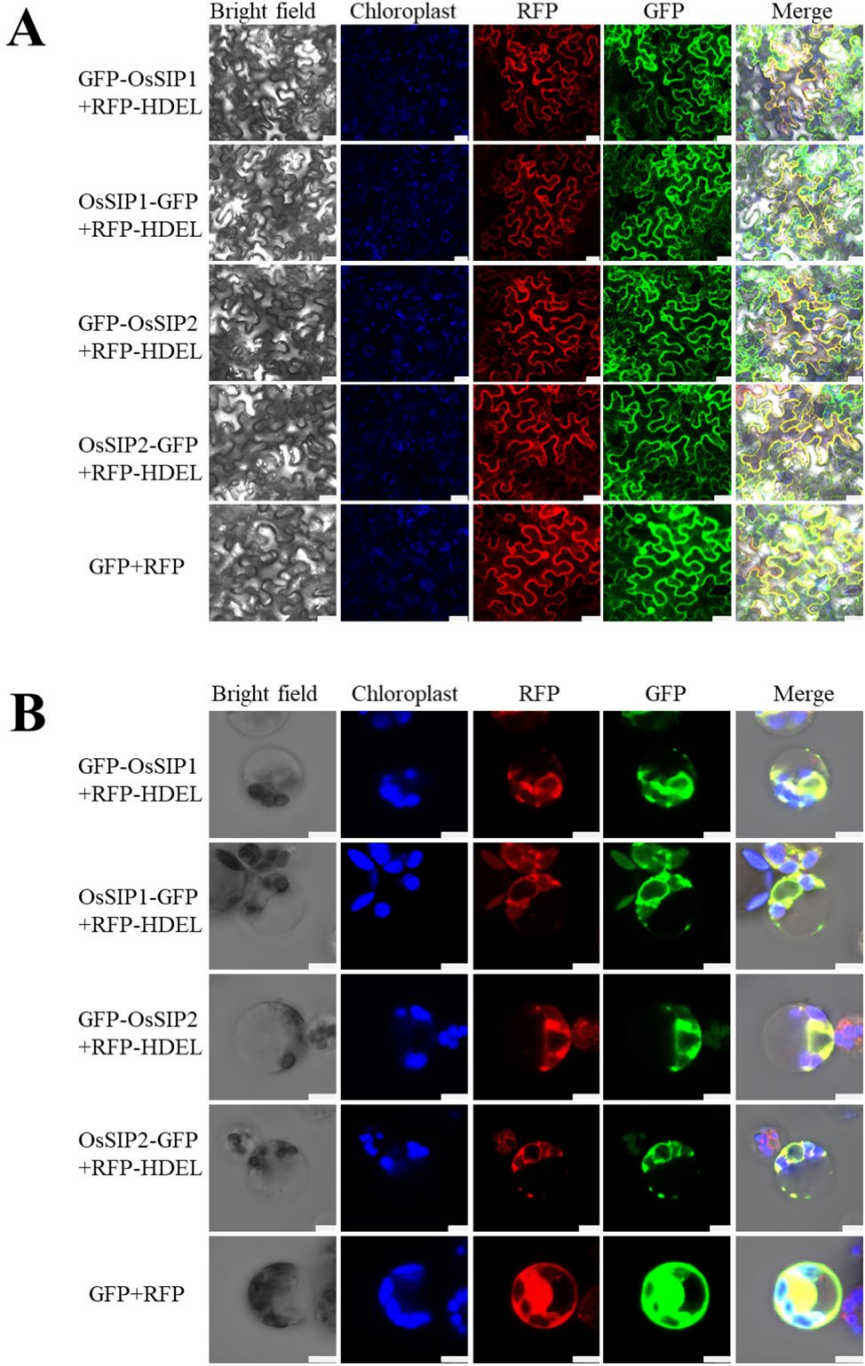
Subcellular co-localization of OsSIP1/2-GFP (C-terminal fusion GFP), GFP-OsSIP1/2 (N-terminal fusion GFP) and free GFP with RFP-HDEL (ER Marker) and free RFP in *N. benthamiana* epidermis (**A**) or rice protoplasts (**B**), driven by the CaMV 35 S promoter. Fluorescence imaging was performed in a Leica SP8 confocal microscope, with a setting for GFP (Ex = 488 nm, Em = 505 to 535 nm), for chlorophyll auto-fluorescence (Ex = 633 nm, Em = 650 to 710 nm) and for RFP (Ex = 532 nm, Em = 550 to 600 nm). Scale bar of figure A/B = 25/5µm.

The heterologous expression in yeast indicates that the ER-localization of OsSIP proteins is not exclusive and that a small amount of the protein is present at the plasma membrane. To investigate the subcellular location of OsSIP1 and OsSIP2 *in planta*, we co-expressed GFP fusion protein of OsSIPs, which were tagged at either the C-terminus or the N-terminus, along with an ER-localized red fluorescence marker (RFP-HDEL) [51] in tobacco epidermal cells (Fig. 4A). Apparent GFP fluorescence signals overlapped with that of ER-RFP, confirming the predominant ER localization of OsSIPs in tobacco epidermal cells (Fig. 4A). Additionally, the fusion of GFP protein to the N-terminus or C-terminus of OsSIPs did not alter their subcellular localization (Fig. 4A). A further experiment was conducted using rice protoplasts isolated from young stem, which gave similar results (Fig. 4B). Due to the low resolution, it was difficult to ascertain whether some SIP proteins were also allocated at the plasma membrane. However, the fluorescent signals suggested that SIP proteins seemed have a broader distribution in the endomembrane system, extended from the ER (Fig. 4B).

### Expression pattern of OsSIP1 and OsSIP2

**Fig. 5:**
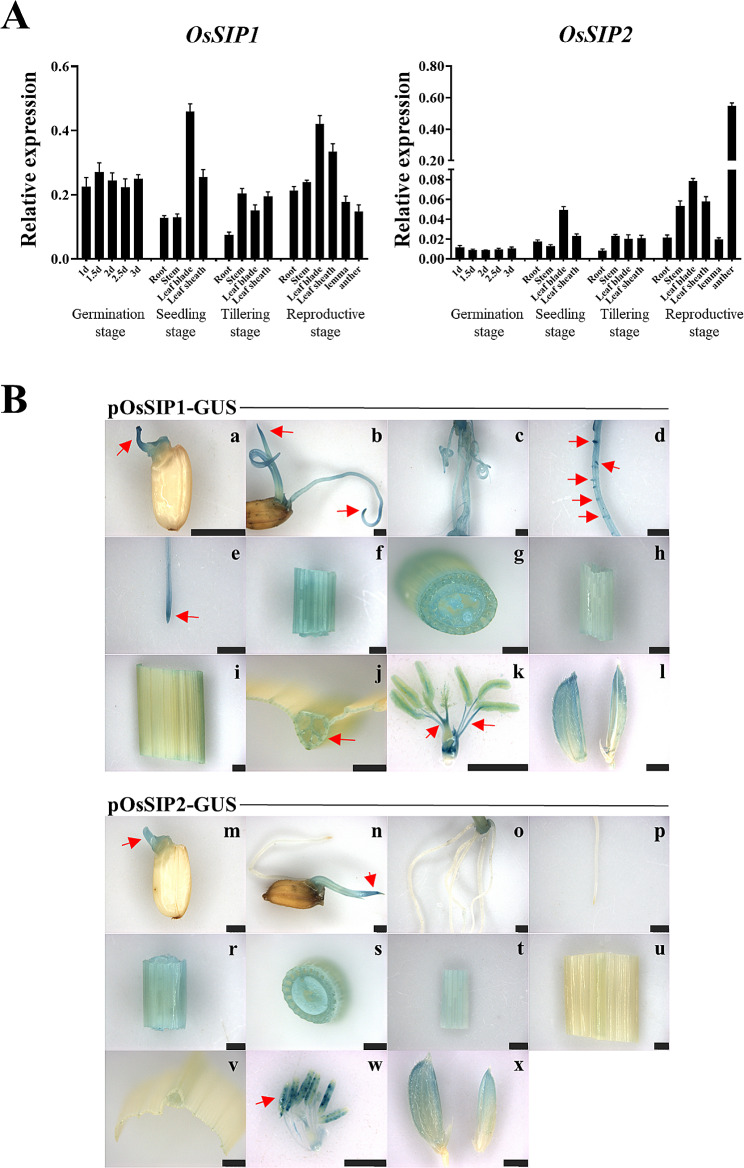
Expression patterns of *OsSIP1* and *OsSIP2* in various tissues of rice (*O. sativa L. cv. Nipponbare*) at different developmental stages. Transcript levels are expressed relative to rice *OsACTIN* in each sample, and values are mean ± SD. Mean and SD values were obtained from three biological replicates and three technical replicates (A). Expression profiles of the *OsSIPs* promoter::*GUS* fusions in *O. sativa L. cv. Nipponbare* (B). pOsSIP1::GUS, (a–l), pOsSIP2::GUS, (m–x). (a, m) Seeds germinated for 2 days; (b, n) Seeds germinated for 4 days; (c, o) adventitious roots; (d)root hair; (e, p) root tip; (f, g, r, s) stem; (h, t) leaf sheath; (i, j, u, v) leaf blade; (k, w) anthers; (l, x) glume. Scale bar = 2.5 mm

Tissue expressing the highest level of *OsSIP1* was found in leaves of seedling and at reproductive stage, with a moderately reduced level at tillering stage. *OsSIP1* transcripts were detected at roughly similar levels in most tissues, but were reduced in the root (Fig. 5A). Much lower expression of *OsSIP2* was found in almost all tissues except the anther, where it reached the maximum transcript levels similar to those of *OsSIP1* in young leaves (Fig. 5A).

To further investigate the tissue-specific expression of *OsSIP1* and *OsSIP2* in rice, we generated transgenic plants expressing the β-glucuronidase (GUS) reporter gene under the control of the native promoters (*pOsSIP1::GUS* and *pOsSIP2::GUS*) (Fig. S1). Histochemical GUS staining was carried out on germinating seeds, 2-week-old seedlings, and reproductive glumes of *pOsSIP1::GUS* and *pOsSIP2::GUS* transgenic plants (homozygous T2 progeny), and the wild-type plant served as a negative control for staining (Fig. S2). It was observed that *OsSIP1* displays stronger GUS signals than *OsSIP2* in all rice tissues tested, except for the anthers. Stronger GUS staining signals were found in both *OsSIP1* and *OsSIP2* at the shoot tips of germinating seeds (Fig. 5B a, m) and at the stem tips of seedlings (Fig. 5B b, n). Additionally, expression of *OsSIP1* was detected in adventitious roots of seedlings (Fig. 5B c), particularly at root hairs and root tips (Fig. 5B d, e). On the contrary, no *OsSIP2* expression signals were detected in the roots (Fig. 5B n, o, p), suggesting that *OsSIP2* was hardly expressed in rice roots. In stem, leaf sheath and glumes, both *OsSIP1* and *OsSIP2* produced visible staining signals, with *OsSIP1* displaying much higher intensity than *OsSIP2* (Fig. 5B f, g, h, l, and r, s, t, x). Moreover, less staining signals were detected in leaf blades and veins for OsSIP1, but not for OsSIP2 (Fig. 5B i, j, u, v). Interestingly, *OsSIP1* expression signal was detected at the base of filaments and pistils (Fig. 5B k), while *OsSIP2* displayed a robust GUS staining signal in anthers (Fig. 5B w). Overall, *OsSIP1* showed variable expression in all tissues, whereas *OsSIP2* showed high expression specific to anthers. The result of the promoter-GUS experiment was in good agreement with that obtained by qRT-PCR detection.

### Expression profiles of *OsSIPs* during biotic and abiotic stresses, as well as exogenous phytohormone treatment

AQPs play a critical role in plant responses to various stressors, including drought, salt, temperature, redox, pathogen attack, as well as hormonal stimuli [4, 15, 20, 21]. To determine the putative cis-acting elements in the promoter of the *OsSIP* genes, analysis of the 2000 bp region upstream of the initiation codon (ATG) of the two *OsSIP* genes was performed using the PlantCARE database (http://bioinformatics.psb.ugent.be/webtools/plantcare/html/). The identified elements are summarized in Table S1. In addition to transcription factor recognition sites, such as those for MYC and MYB, a wide range of light-responsive elements, meristematic expression elements (CAT-box, CCGTCC-box), gibberellin-responsive elements (P-box, TATC-box), anaerobic induction elements (ARE), drought stress-responsive elements (MBS), and several stress-responsive elements (STRE, WRE3, W-box) are anticipated in the promoters of both *OsSIPs* genes. The promoter of the *OsSIP1* gene also contains response elements for a salicylic acid (SA), methyl jasmonate (MeJA), ethylene, and low temperature (LTR). Similarly, the promoter of the *OsSIP2* gene shows the presence of response elements for abscisic acid (ABA) and auxin (Table S1). Overall, *OsSIP1* contains a greater number of response elements in its promoter than *OsSIP2*.

**Fig. 6:**
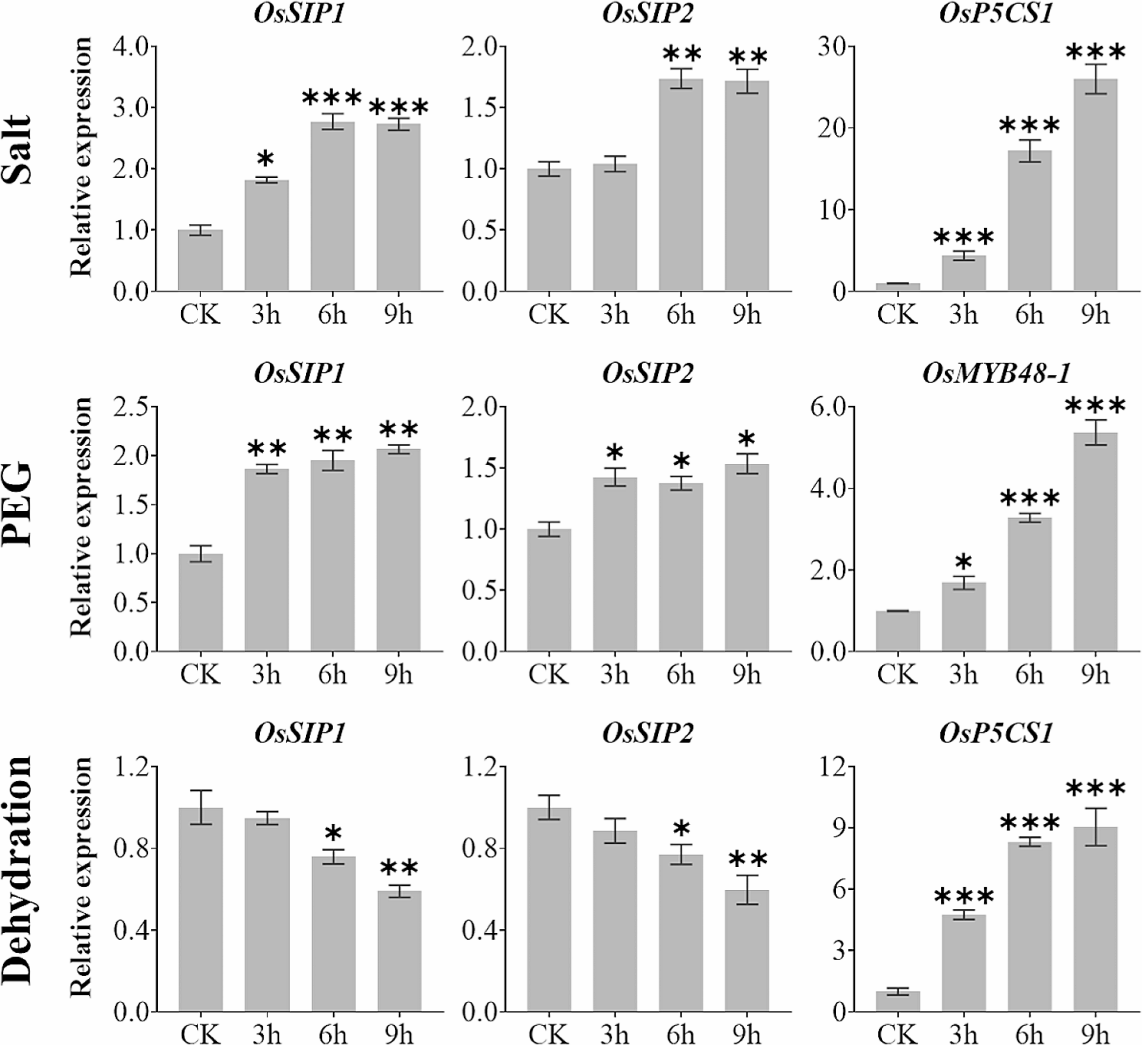
Expression analysis of *OsSIP1*, *OsSIP2* with stress response marker genes *OsP5CS1* (salt, dehydration) and *OsMYB48-1* (osmotic) under salt, osmotic and dehydration stress treatments. Two-weeks-old rice seedlings (*O. sativa L. cv. Nipponbare*) were treated separately with salt stress (150 mM NaCl), osmotic stress (20% PEG6000) and dehydration (on filter paper without water supply). Whole plants were collected for qRT-PCR analysis after 3, 6, and 9 h treatments. Values represent the mean ± SD of three biological replicates and three technical replicates, and the Y-axis represents the relative expression level normalized to *OsACTIN*. Asterisks indicate significant differences (*P < 0.05, **P < 0.01, and ***P < 0.001) based on Student t-test compared to CK (0 h time point without treatment)

**Fig. 7:**
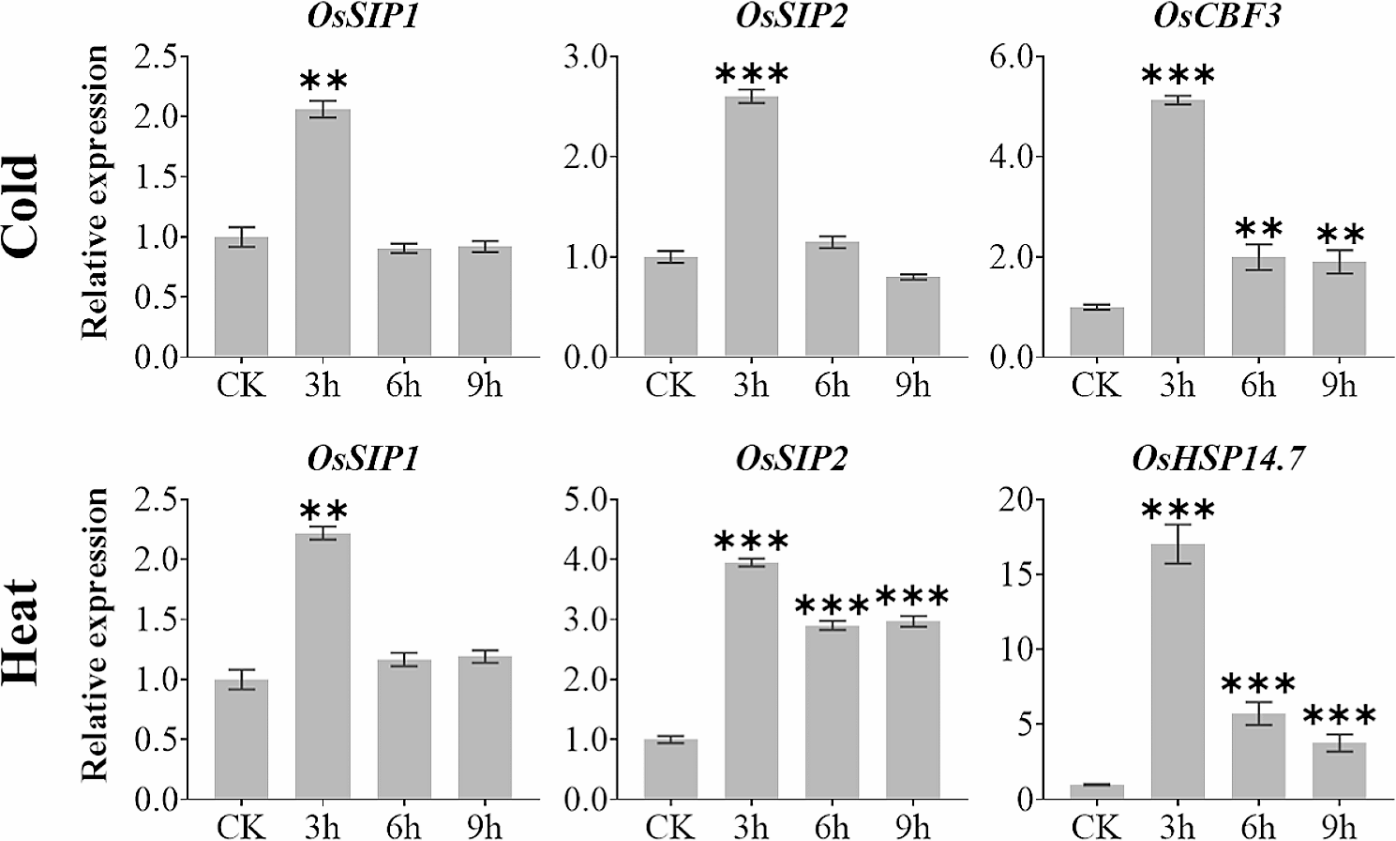
Expression analysis of *OsSIP1*, *OsSIP2* with temperature response marker genes *OsCFB3* (cold) and *OsHSP14.7* (heat) under extreme temperature stress treatment. Two-weeks-old rice seedlings (*O. sativa L. cv. Nipponbare*) were treated separately at 4 °C or 42 °C. Whole plants were collected for qRT-PCR analysis after 3, 6, and 9 h treatments. Values represent the mean ± SD of three biological replicates and three technical replicates, and the Y-axis represents the relative expression level normalized to *OsACTIN*. Asterisks indicate significant differences (**P < 0.01, and ***P < 0.001) based on Student t-test compared to CK (0 h time point without treatment)

**Fig. 8:**
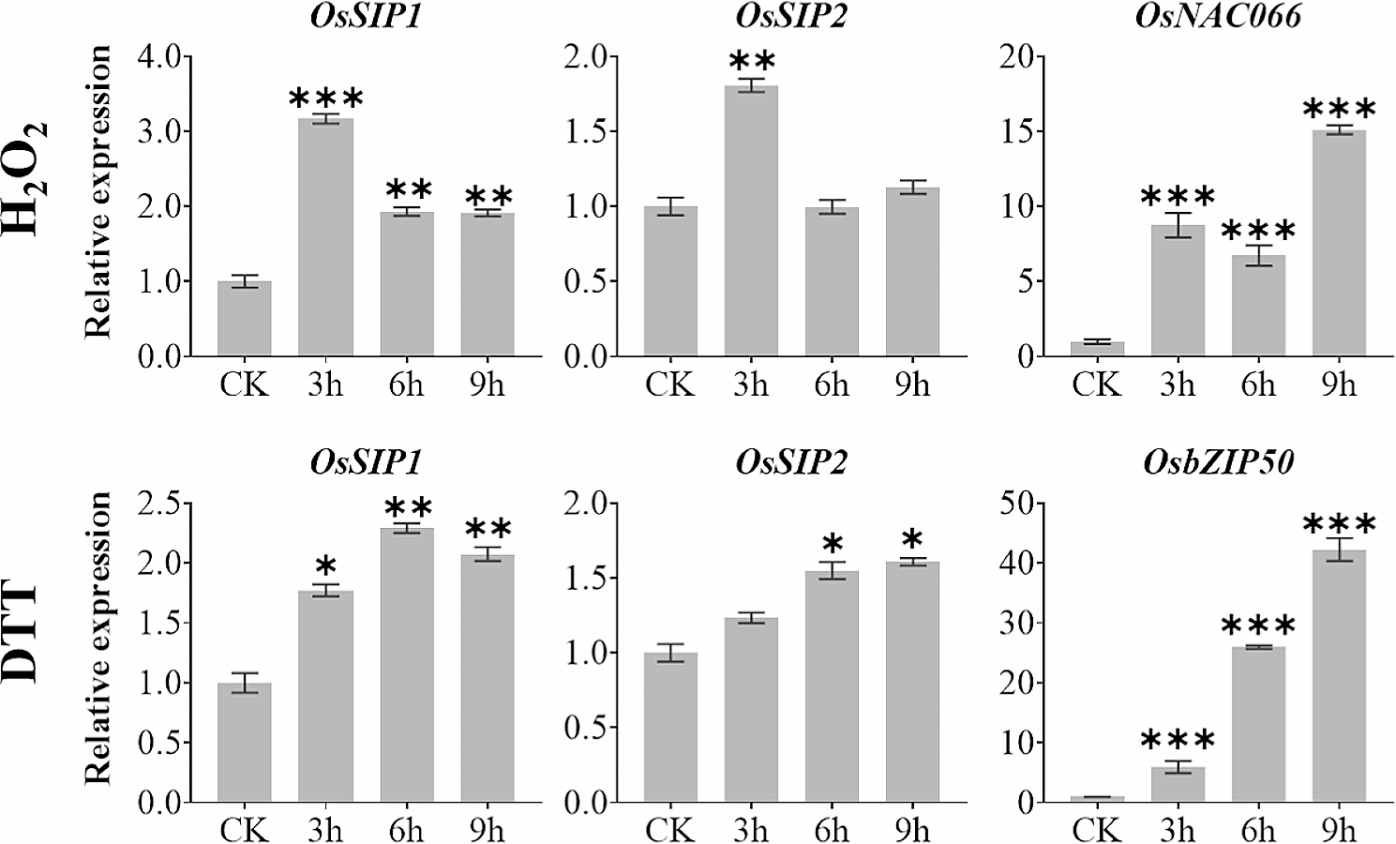
Expression analysis of *OsSIP1*, *OsSIP2* with stress response marker genes *OsNAC066* (oxidative) and *OsbZIP50* (ER stress) under oxidative and ER stress treatments. Two-weeks-old rice (*O. sativa L. cv. Nipponbare*) seedlings were treated separately with 1% H_2_O2 or 2 mM DTT (dithiothreitol). Whole plants were collected for qRT-PCR analysis after 3, 6, and 9 h treatments. Values represent the mean ± SD of three biological replicates and three technical replicates, and the Y-axis represents the relative expression level normalized to *OsACTIN*. Asterisks indicate significant differences (*P < 0.05, **P < 0.01, and ***P < 0.001) based on Student t-test compared to CK (0 h time point without treatment)

We then carried out experiments using 2-week-old rice seedlings challenged by salt and osmotic stresses and demonstrated that both *OsSIP1* and *OsSIP2* were up-regulated (Fig. 6). Nevertheless, the expression of *OsSIP1* and *OsSIP2* was significantly diminished in the condition where water was withdrawn to cause dehydration. *OsP5CS1* and *OsMYB48-1*, which are acknowledged to respond positively to dehydration, salt stress, and osmotic stress [52, 53], were utilized as treatment controls (Fig. 6). On the other hand, cold and high temperature stresses caused a shift in expression of *OsSIP1* and *OsSIP2* during the first three hours after treatment, which was similar to that of the known stress-responsive marker genes, namely *OsCBF3* and *OsHSP14.7* [54, 55] (Fig. 7). Additionally, oxidative stress imposed by hydrogen peroxide treatment up-regulated the expression of *OsSIP1* and *OsSIP2* as well as an oxidative stress marker gene *OsNAC066* [56] (Fig. 8). Similarly, under DTT-induced ER stress, the expression of both *OsSIP1* and *OsSIP2* were slightly increased, in contrast to the strong response of the marker gene *OsbZIP50* [57] (Fig. 8). It was concluded that, in terms of quantitative comparison, *OsSIP1* was most responsive to H_2_O_2_ and salt treatments while *OsSIP2* to heat and DTT stresses.

When seedlings were treated with gibberellin (GA), abscisic acid (ABA), methyl jasmonate (MeJA) or salicylic acid (SA), both *OsSIP1* and *OsSIP2* showed up-regulated expression to some extent (Fig. S3). We also checked the expression of the rice GA synthesis gene *OsGA20ox2* [58], and marker genes for hormone signalling response, namely *OsABF1*, *OsLOX2* and *X58877* [59–61] for ABA, MeJA and SA, under the same conditions (Fig. S3). These results suggest that *OsSIP1* and *OsSIP2* are little regulated by the tested phytohormone signalling pathways.

**Fig. 9:**
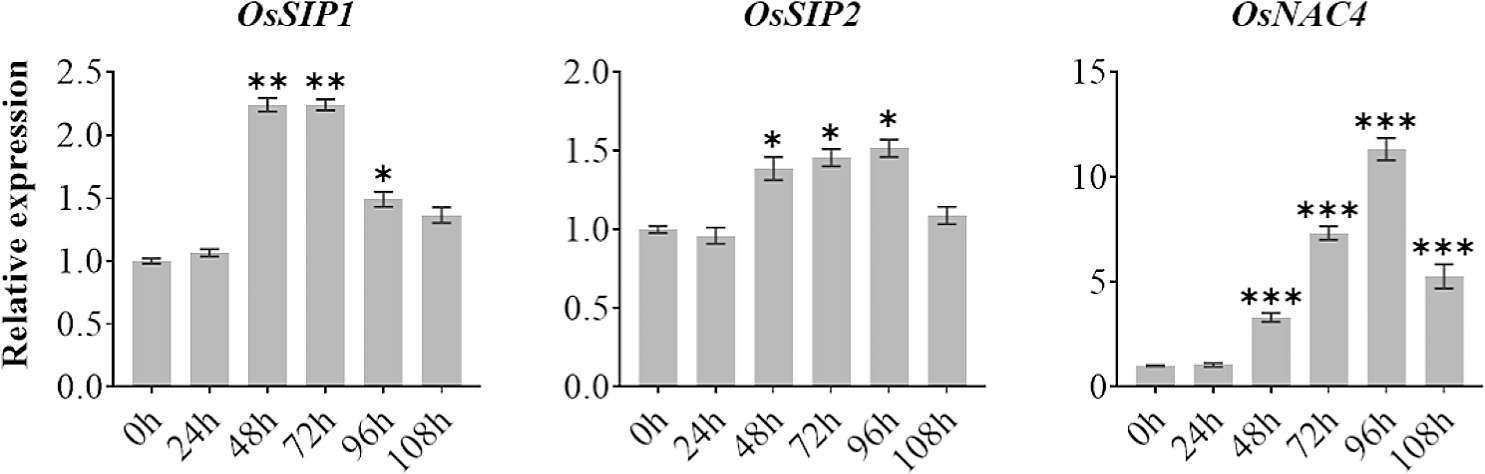
Expression analysis of two *OsSIP* genes and the defence-related marker gene *OsNAC4* in response to *M. oryzae* infection. qRT-PCR analysis of *OsSIP1*, *OsSIP2* and *OsNAC4* in rice (*O. sativa L. cv. Nipponbare*) at 0, 24, 48, 72, 96, and 108 h after pathogen treatment. The Y-axis represents the relative expression level normalized to *OsACTIN*. Three biological replicates and three technical replicates were used. Asterisks indicate significant differences (*P < 0.05, **P < 0.01, and ***P < 0.001) based on Student t-test compared to 0 h

Finally, to investigate the possible involvement of *OsSIPs* genes in the response to pathogen attack, we spray-inoculated rice seedlings at the three-leaf stage with *Magnaporthe oryzae* strain Guy11 and collected samples of diseased leaves every 24 h over a period of 5 d (Fig. 9). Both *OsSIP1* and *OsSIP2* showed moderate up-regulation in response to *M. oryzae* infection, with *OsSIP1* being stronger. The *OsNAC4* gene, used as a control marker, showed increased transcript levels in the infected leaves (Fig. 9).

## Discussion

From an evolutionary point of view, SIP-like aquaporins are present in vascular plants from mosses to angiosperms, whereas the appearance of SIP2s in angiosperms came later [17, 23–25, 62]. A recent study, analyzing AQPs genes in eleven wild and cultivated rice species, has proposed that the SIP-like aquaporins are likely the ancestral members of all AQPs in land plants [63]. We found that *OsSIP1* had a much broader expression pattern across tissues than *OsSIP2*, which was preferentially expressed in anthers (Fig. 5). Both OsSIP1 and OsSIP2 were similarly regulated at varying levels by a number of abiotic and biotic stresses, and hormone treatments (Figs. 6, 7 and 8, S3), hinting at the existence of a common and conserved mechanism to control their functionality. However, it is plausible that OsSIP1 is more impressive due to its higher and broader expression, greater induction by H_2_O_2_ and salt, and stronger response to pathogen (Figs. 6, 7, 8 and 9). Thus, the two members of the SIP subfamily in rice are likely to have similar but distinct functions in growth and development, as well as in stress resistance.

The pronounced anther expression preference of *OsSIP2* suggests that, like AtSIP2, it may be involved in reproduction [26]. Previous research has shown that *AtSIP1;1* and *AtSIP2;1* are highly expressed in *Arabidopsis* pollen [24]. Knockout of *AtSIP2;1* in *Arabidopsis* resulted in a significant reduction in pollen germination, and pollen tube elongation was significantly lower in the *atsip2;1* mutant than in the wild type [26]. The *Arabidopsis* double knockout mutant *sip1;1 pip1;2* shows comparable defects in pollen hydration to *atsip2;1*, and together they regulate the successful completion of pollen hydration in the pistil [27].

At present, it is not possible to assign a clear function to the intracellularly localized OsSIPs, but they do facilitate the passage of water and H_2_O_2_ through the plasma membrane, and even very weak (OsSIP1) to no (OsSIP2) passage of methylamine, when partially distributed on the cell surface, as shown by heterologous expression in yeast mutant cells (Fig. 3). Previous stopped-flow experiments using isolated ER membrane vesicles from yeast cells expressing *AtSIP1;1*, *AtSIP1;2*, *AtSIP2;1*, and *VvSIP1;1* also demonstrate that the SIP1-likes but not SIP2 are water permeable channel, enabling 1.3 to 2.5 folds faster water conductance than the empty vesicles. Solute like glycerol, ammonia, sorbitol, and urea are not good permanents for SIPs [24, 28].

In terms of the selectivity filter in SIP channels, the two NPA motifs are quite conserved, but the ar/R regions somehow differ between SIP1 and SIP2 groups in that the later usually contains a positive charge amino acid residue (H) and two hydrophilic residues (S and G) (Fig. S4). In cases of OsSIP1 and OsSIP2, the four residues of the ar/R region are S55, V180, P189, and N195 for OsSIP1, and L59, H179, G188, and S194 for OsSIP2. Consequently, OsSIP1 has a wider pore diameter than OsSIP2 (Fig. 2E). That may explain in part why OsSIP2 is not permeable for methylamine.

A recent study has demonstrated that the OsRINGzf1 RING-H2-type E3 ligase has a positive impact on drought tolerance in rice by promoting the degradation of OsPIP2;1, and OsSIP1 is also an ubiquitinated substrate by OsRINGzf1 [64]. Nevertheless, the exact role of OsSIP1 and OsSIP2 play in water homeostasis maintenance and the regulation of their function in rice requires further investigations.

## Conclusion

OsSIP1 and OsSIP2 form the two-member Small Intrinsic Proteins subfamily of aquaporins in rice, they conduct water and H_2_O_2_. Moreover, OsSIP1 has a weak ability to conduct methylamine while OsSIP2 does not. Both proteins are mainly localized to the ER, although transient localization to the plasma membrane is not excluded. OsSIP1 is widely expressed in most tissues and during developmental stages, while OsSIP2 expressed preferentially in the anther. Both genes are up-regulated under a variety of stress conditions and by different hormone treatments, except that dehydration moderately reduces their expression. Taken together, this study provides basic information to further decipher the potential roles of the plant SIP-like proteins.

### Electronic supplementary material

Below is the link to the electronic supplementary material.

Supplementary Material 1

## Data Availability

All data generated or analyzed during this study are included in this published article and its supplementary information files.

## Abbreviations

AQPs: Aquaporins
ER: Endoplasmic reticulum
TM: Transmembrane
LA-LC: Loop regions A-C
MIPs: Major intrinsic proteins
PIPs: Plasma membrane intrinsic proteins
TIPs: Tonoplast intrinsic proteins
NIPs: Nod26-like intrinsic proteins
SIPs: Small and basic intrinsic proteins
GIPs: GlpF-like intrinsic proteins
XIPs: Uncategorized X intrinsic proteins
HIPs: Hybrid intrinsic proteins
NPA: Asparagine-proline-alanine
ar/R: Aromatic-arginine
CO_2_: Carbon dioxide
H_2_O_2_: Hydrogen peroxide
NH_3_: Ammonia
KCl: Potassium chloride
MeA: Methylamine
SDS-PAGE: Sodium dodecyl sulfate polyacrylamide gel electrophoresis
CBB: Coomassie brilliant blue
GUS: β-glucuronidase
GFP: Green fluorescent protein
RFP: Red fluorescent protein
DTT: Dithiothreitol
GA: Gibberellin
ABA: Abscisic acid
MeJa: Methyl jasmonate
SA: Salicylic acid
qRT-PCR: Quantitative real-time polymerase chain reaction
