# Supplementary material for: Characterization of SIPs-type aquaporins and their roles in response to environmental cues in rice (Oryza sativa L.)
Source: BMC Plant Biol. 2024 Apr 22;24:305. doi: 10.1186/s12870-024-05002-x (PMC11034084; doi:10.1186/s12870-024-05002-x)
Supplement: Supplementary file 1 — Supplementary Material 1 [file 12870_2024_5002_MOESM1_ESM.pdf]

Supplementary materials

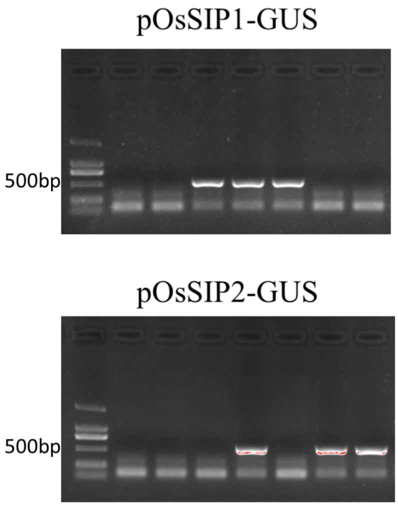

Fig. S1 Identification of *OsSIPs* promoter-GUS positive transgenic lines in rice at the genomic level

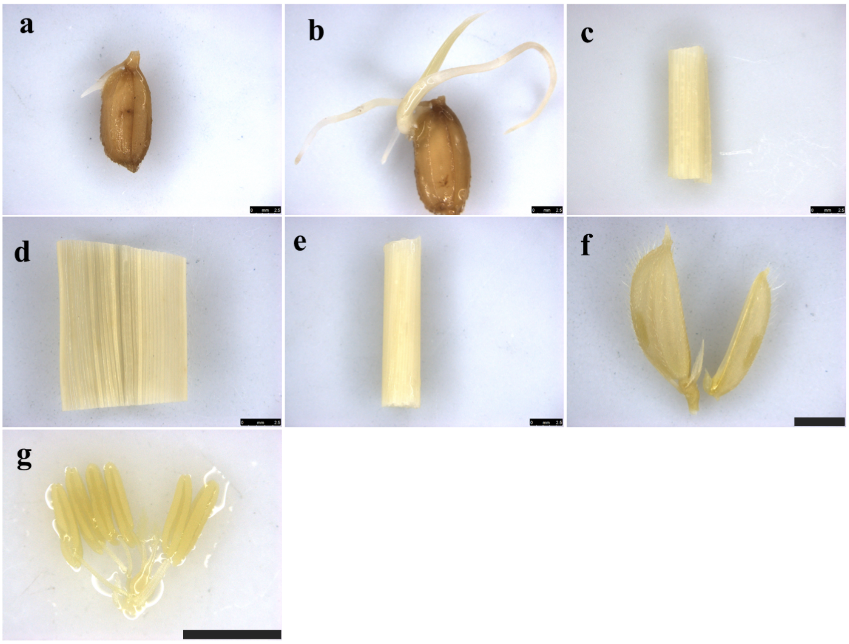

Fig. S2 Negative control of GUS staining in rice (*O. sativa L. cv. Nipponbare*). (a) seed germinated for 2 days; (b) seed germinated for 4 days; (c) leaf sheath; (d) leaf blade; (e) stem; (f) glume; (g) anthers. Scale bar=2.5mm

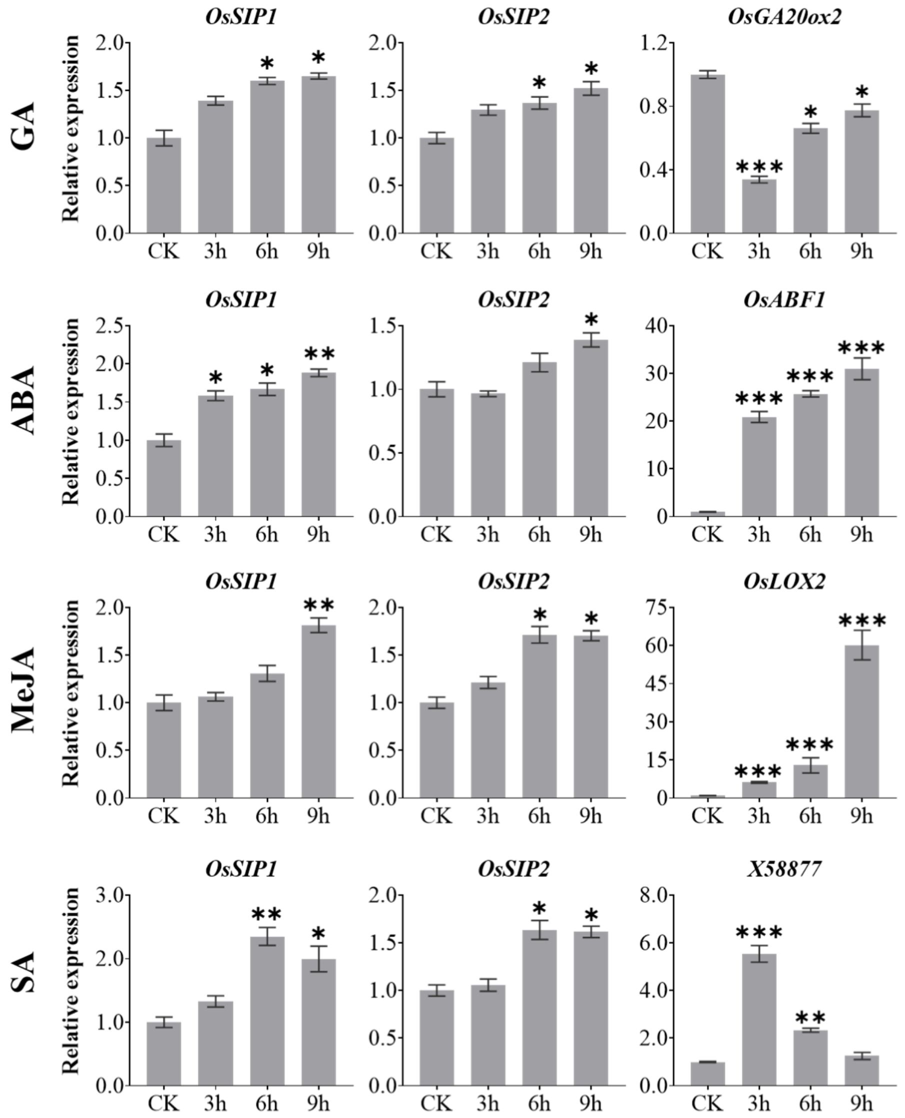

Fig. S3 Expression analysis of *OsSIP1*, *OsSIP2* with hormone response marker genes *OsGA20ox2* (GA), *OsABF1* (ABA), *OsLOX2* (MeJA) and *X58877* (SA) under different hormone treatments. The 2-week-old rice seedlings (*O. sativa L. cv. Nipponbare*) were treated with 100 μM concentrations of GA, ABA, MeJA, and SA separately. Whole plants were collected for qRT-PCR analysis after 3, 6, and 9 h treatments. Values represent the mean ± SD of three biological replicates and three technical replicates, and the Y-axis represents the relative expression level normalized to *OsACTIN*. Asterisks indicate significant differences (**P* < 0.05, ***P* < 0.01, and ****P* < 0.001) based on Student t-test compared to CK (0 h time point without treatment).

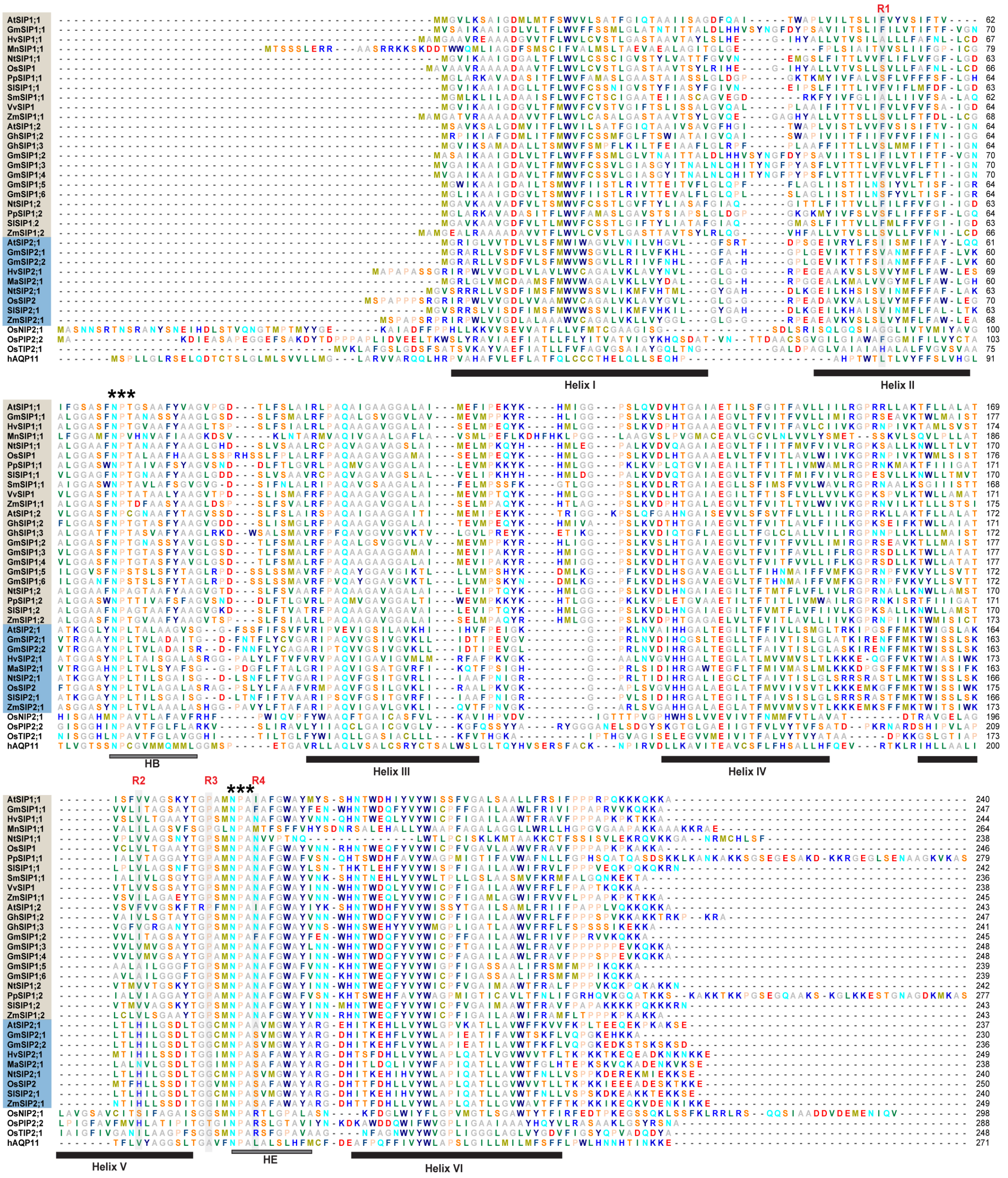

Fig. S4 Multiple sequence comparison of the SIP family from *Oryza sativa* (Os), *Arabidopsis thaliana* (At), *Vitis vinifera* (Vv), *Zea mays* (Zm), *Nicotiana benthamiana* (Nt), *Selaginella moellendorffii* (Sm), *Physcomitrium patens* (Pp), *Monoraphidium neglectum* (Mn), *Solanum lycopersicum* (Sl), *Gossypium hirsutum* (Gh), *Hordeum vulgare* (Hv), *Glycine max* (Gm), *Musa acuminata* (Ma) and *Homo sapiens* (hAQP11) respectively. Asterisks indicate NPA regions. R1-R4 on top of the sequences denotes the four residues of the ar/R constriction. The six transmembrane helices are indicated below the sequences. SIP1s and SIP2s are highlighted with yellow and blue background, respectively.

Table S1 Cis-elements in the promoters of rice *SIP* genes.*

Promoter prediction by PlantCARE

	CAT-box	CCGTCC-box	as-1	STRE	ARE	WRE3	MBS	LTR	ERE	W-box	TCA-element	TGACG-motif	TATC-box	P-box	ABRE	AuxRR-core	MYB	MYC	light responsive
Ossip1	1	2	1	3	1	1	1	1	1	1	1	1	--	1	--	--	8	5	8
Ossip2	1	--	--	3	1	1	1	--	--	1	--	--	1	2	2	1	9	6	3

* Promoter regions of 2000 bp upstream of *OsSIP1* and *OsSIP2* were analyzed using the PLACE program. CAT box, CCGTCC box： meristematic expression elements; as-1, TCA element: SA responsive element; ARE: anaerobic induction elements; WRE3: stress-responsive element; MBS: MYB transcription factors binding site involved in drought inducibility; LTR: low temperature responsive element; ERE: ethylene responsive element; W-box: WRKY transcription factor binding site; TGACG motif: MeJA responsive element; TATC-box, P-box: gibberellin responsive element; ABRE: abscisic acid responsive elements; AuxRR-core: auxin responsive element; MYB, MYC: MYB and MYC transcription factor recognition sites;

Table S2 List of primers used in this study.

	**Primer id**	**Primer Sequence (5’- 3’)**
Gateway cloning	OsSIP1-F	CACCATGGCGGTGGCGGCGGTG
	OsSIP1-R	TGCTTTCTTGGCCTTAGGTTTAGGTG
	OsSIP2-F	CACCATGTCGCCCGCTCCGCCGCCGTC
	OsSIP2-R	CTCCTTCTTGGTTTTGCTTTCATCTGC
	Promoter-OsSIP1-F	CACCCTGACAGTAGAAGCGATCCGAGCCATTG
	Promoter-OsSIP1-R	ATCGCCGCAGCCGCCGCTC
	Promoter-OsSIP2-F	CACCTCCCTCACCTCGTTTGATCTCCTCCTGGAC
	Promoter-OsSIP2-R	GGCCTGACCGGTGGGGGGGAGGGGAGGTA
Yeast	pRS-OsSIP1-F	GGATCCATGGCGGTGGCGGCGGTG

expression vectors	pRS-OsSIP2-F	GGATCCATGTCGCCCGCTCCGCCGCCGTC
	pRS-GFP-F	GGATCCATGGTGAGCAAGGGCGAGGAG
	pRS-GFP-R	GAATTCCTTGTACAGCTCGTCCATGCCGAGAG
Transgenic Plant Screening	Hyg-F	CGAGAGCCTGACCTATTGCAT
	Hyg-R	CTGCTCCATACAAGCCAACCAC
RT-qPCR	OsSIP1-F	AATCGCCACAACACATGGGA
	OsSIP1-R	TTAGGTTTAGGTGCCGGTGG
	OsSIP2-F	TCATGAACCCTGCATCTGCTT
	OsSIP2-R	AAGGTTGCTTGGAGGGGTG
	OsNAC4- F	TCCTGCCACCATTCTGAGATG
	OsNAC4- R	TTGCAGAATCATGCTTGCCAG
	Actin-F	TCCATCTTGGCATCTCTCAG
	Actin-R	GTACCCTCATCAGGCATCTG
	OsABF1-F	GCATGATCAAGAACCGTGAGT
	OsABF1-R	GAACCGTCTTCTCTGCCTCTT
	OsX58877-F	TGCCAGGGAAAGCAACAATT
	OsX58877-R	GACGCACTGTCATAGTCTGCCTAA
	OsGA20ox2- F	CCAATTTTGGACCCTACCGC
	OsGA20ox2- R	GAGAGAAGCCCAACCCAACC
	OsLOX2-F	GCATCCCCAACAGCACATC
	OsLOX2-R	AATAAAGATTTGGGAGTGACATATTGG
	OsbZIP50-F	TGCCATGAAATCGAGGGAGAG
	OsbZIP50-R	AGCCAAAGCAGGGAAACCAG
	OsHSP17.4-F	AGGTCAAGGTGGAGGTTGAG
	OsHSP17.4-R	CCACTTGTCCGTCTTCTCCT
	OsP5CS1-F	GCTGACATGGATATGGCAAAAC
	OsP5CS1-R	GTAAGGTCTCCATTGCATTGCA

	OsMYB48-1-F	TGGGATTTCTTAGCAAAGGTGTC
	OsMYB48-1-R	ACACACCACCCATACACACGAC
	OsNAC066-F	TGCATGCAAGAAGCTGAGGTCTG
	OsNAC066-R	TGCTGCTTCCTGTAGGTGATGC
	OsCBF3-F	ATCAAGCAGGAGATGAGCGG
	OsCBF3-R	GTCTCCCTGAACTTGGTCCG
